# Classification of Power Facility Point Clouds from Unmanned Aerial Vehicles Based on Adaboost and Topological Constraints

**DOI:** 10.3390/s19214717

**Published:** 2019-10-30

**Authors:** Yuxuan Liu, Mitko Aleksandrov, Sisi Zlatanova, Junjun Zhang, Fan Mo, Xiaojian Chen

**Affiliations:** 1 School of Remote Sensing and Information Engineering, Wuhan University, Wuhan 430079, China; liuyx@whu.edu.cn; 2 Department of Built Environment, University of New South Wales, Sydney 2052, Australia; Mitko.aleksandrov@unsw.edu.au (M.A.); s.zlatanova@unsw.edu.au (S.Z.); 3 Beijing New3S Technology Pty. Ltd., Beijing 100085, China; 4 Land Satellite Remote Sensing Application Centre, MNR, Beijing 100048, China; 5 Faculty of Business Administration, The Chinese University of Hong Kong, Hong Kong 999077, China; chenxiaojian@link.cuhk.edu.hk

**Keywords:** point cloud classification, power line, power tower, the Adaboost algorithm, topological constraint

## Abstract

Machine learning algorithms can be well suited to LiDAR point cloud classification, but when they are applied to the point cloud classification of power facilities, many problems such as a large number of computational features and low computational efficiency can be encountered. To solve these problems, this paper proposes the use of the Adaboost algorithm and different topological constraints. For different objects, the top five features with the best discrimination are selected and combined into a strong classifier by the Adaboost algorithm, where coarse classification is performed. For power transmission lines, the optimum scales are selected automatically, and the coarse classification results are refined. For power towers, it is difficult to distinguish the tower from vegetation points by only using spatial features due to the similarity of their proposed key features. Therefore, the topological relationship between the power line and power tower is introduced to distinguish the power tower from vegetation points. The experimental results show that the classification of power transmission lines and power towers by our method can achieve the accuracy of manual classification results and even be more efficient.

## 1. Introduction

In recent years, with the development of economic construction, the demand for electricity has increased rapidly, thereby increasing the demand for power grid construction. Managing large power networks to effectively ensure the normal operation of the power grid and the safe transmission of electricity is clearly important. In order to ensure the safety of transmission lines and prevent the occurrence of power grid accidents, power departments need to conduct periodic safety inspections of power lines [1].

Traditional power line inspection work is mainly carried out by electricians. There are some shortcomings of such work including the high intensity of operation, long operation period, inaccurate data acquisition, low accuracy, low reuse, and difficulty of working in complex terrain areas. Researchers have proposed the use of various remote sensing approaches such as synthetic aperture radar (SAR) [2,3], thermal images [4], optical images [5,6], airborne laser scanning (ALS) [7,8], and unmanned aerial vehicle (UAV) [9] to identify power facilities. Matikainen et al. [10] give a detailed overview of different sensors in the application of power facility detection. With the maturity of drone technology and the enhancement of light detection and ranging (LiDAR) point cloud processing ability, UAV LiDAR technology is increasingly being used in power line inspection [11]. Compared to other sensors, UAV LiDAR has the characteristics of convenience, speed, and accuracy, saving significant time and human resources. The basic role of point clouds in power line inspection is to determine the various objects in the transmission corridor, and use the relationship between the various objects to check the security risks.

The quantity of UAV LiDAR data is large, and achieving quality requirements is different due to complex surface shapes, creating difficulties in the automatic processing of data in later stages of analysis. In terms of power line inspection, the accurate classification of power lines and power towers is the key factor affecting the final results. Qin et al. [12] developed a new LiDAR data collection method using cable inspection robot (CIR), and proposed corresponding power line point extraction methods based on the CIR data. They first extracted the crude result by using the position and orientation system (POS), and then refined the initial result using structured partition. Aiming at extracting transmission lines from multiple terrains, Shen et al. [13] proposed using different height thresholds to classify points of ground objects and transmission towers, where the thresholds were based on the geography of overhead transmission corridors and the data structure of airborne LiDAR point clouds of transmission lines. Lodha et al. [14] used the Adaboost algorithm to classify the airborne LiDAR data, achieving high classification accuracy. However, the process needs to extract features from corresponding images, and the accurate registration of images and airborne LiDAR power line point clouds, making the process challenging. Kim et al. [15] used random forests for power line classification. Although they obtained relatively high objective classification results, it was difficult to process power lines at multiple scales. Guo et al. [16] extracted 26 features and used JointBoost to create a classifier with five main classes: building, ground, vegetation, power lines, and pylons, obtaining high-quality classification results.

Machine learning-based methods have achieved good performance, but they need to extract many features first, which reduces the efficiency. In this paper, in order to use simple features to classify point cloud data and meet the data processing requirements of power line inspection, two sets of simple 2D and 3D features are designed based on the characteristics of power line cloud data. For each kind of object, only the top five features with the best distinguishing ability are selected, where different weights are given to these features automatically using the Adaboost algorithm [17,18,19] to form a strong classifier. Coarse classification results are obtained using the classification model. Due to the overlap of the selected key features, and particularly the total overlap of power tower and vegetation features, the coarse result has many wrongly classified points. To improve classification accuracy, we used the spatial characteristics of power lines and the topological relationship between power lines and power towers to refine the coarse result, obtaining more accurate classification results.

The rest of this paper is organized as follows. Section 2 defines the proposed 2D and 3D features. Section 3 describes our proposed method in detail. Section 4 presents the experiments and discussion. Section 5 concludes the paper.

## 2. Data Preprocessing and Feature Extraction

**Figure 1:**
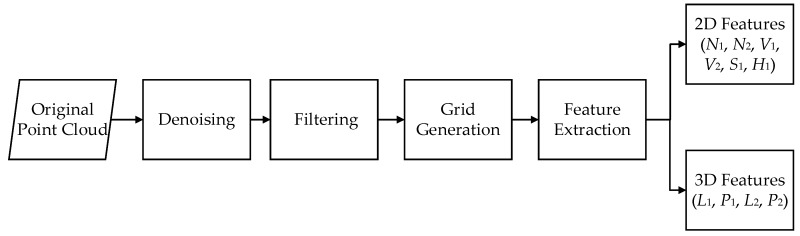
The workflow of feature extraction.

Before feature extraction, the point cloud data are first denoised, and then filtered to obtain the ground topographic information. The processing flow, integrating six steps, which is shown in Figure 1.

### 2.1. Denoising and Filtering

The isolated points, low points, and high points that exist in an airborne laser point cloud represent noise that is difficult to avoid during point cloud data collection. Moreover, the existence of such points will cause large errors in key feature extraction. Therefore, a denoising process should be conducted before point cloud data classification. Statistical methods can be adopted to denoise these point cloud. The three-dimensional grid is used to divide point cloud data. The number of points that fall into each grid is summarized, and whether there are points in surrounding grids is determined. If the number of points in the grid of a point is smaller than a certain amount and there is no point in its surrounding grids, this point is determined to be noise.

The ground, as the basis of power facilities, plays a key role in the classification process. In order to obtain the characteristics of the terrain, it is filtered by the recursive terrain filter developed by Sohn and Dowman [20], and the basic terrain scene is constructed, providing reference for the lowest grids in calculating grid features.

### 2.2. Data Feature Extraction

The point cloud reflects the morphological characteristics of the three-dimensional (3D) space of ground objects, and different ground objects have different local characteristics. Since the classification of points in a small local area is identical, two different point cloud division modes are adopted in this paper. The two-dimensional grid is used to calculate the plane and vertical distribution features of the points in the neighborhood of the grid, and the three-dimensional grid is used to calculate the features of the neighboring points in 3D space.

#### 2.2.1. 2D Grid Features

**Figure 2:**
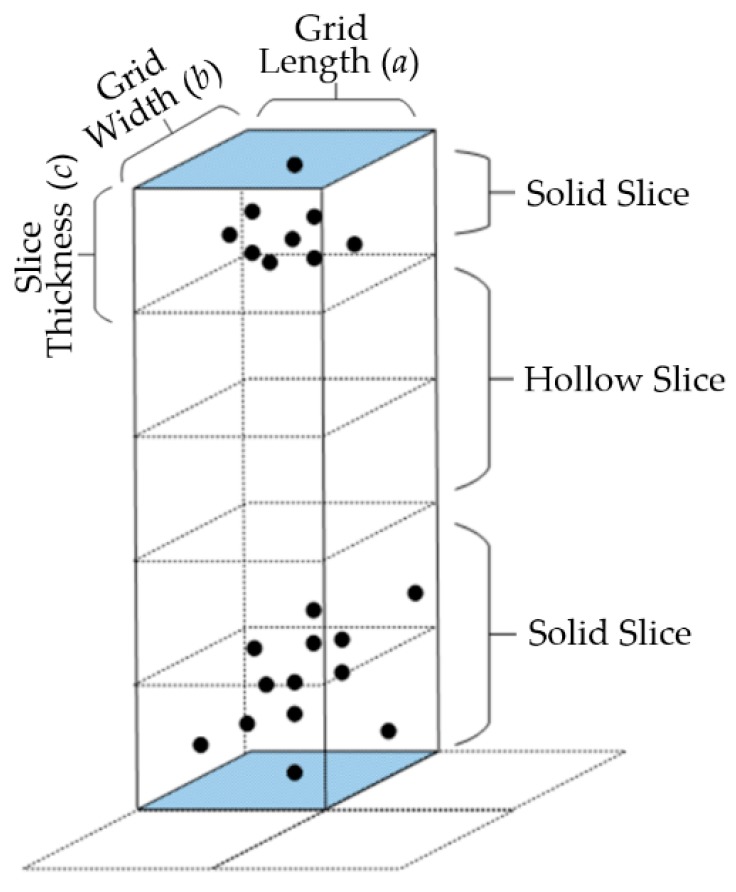
Vertical space slice.

The number of solid slices ($N_{1}$) and number of hollow slices ($N_{2}$) in vertical space: As shown in Figure 2, the area between the highest and lowest points is sliced according to a certain thickness in the two-dimensional grid. Slices with points are solid slices, and slices without points are hollow slices. The number of slices in the vertical space shows the stratification state and dispersion state of the point cloud in the local space.

Vacancy rate ($V_{1}$) and vacancy rate under points ($V_{2}$): The vacancy rate is defined as the ratio between the number of hollow slices and the total number of hollow and solid slices. The vacancy rate represents the point cloud stratification degree in local space. The higher the vacancy rate, greater the proportion of hollow slices in vertical space, and the higher the point stratification and dispersion degree in vertical space. The vacancy rate under points means the ratio of the number of hollow slices under the layer of points to the number of layers where the points exist. The value of the vacancy rate can basically show the distribution location of the stratified point cloud in the local area.

Normalized altitude ($H_{1}$): Normalized altitude [21] refers to the altitude after subtracting the height of the lowest point in each grid from the points in all the grids. Normalized altitude represents the dispersion of points in the local area of the grid.

Altitude jump ($S_{1}$): Non-terrain points, such as the points of the top of a building, are often higher than the surrounding ground points. In addition, obvious vertical altitude jumps with a ladder pattern exist in some construction edges, and appear in pairs in the grids where the line passes through the construction [21].

**Figure 3:**
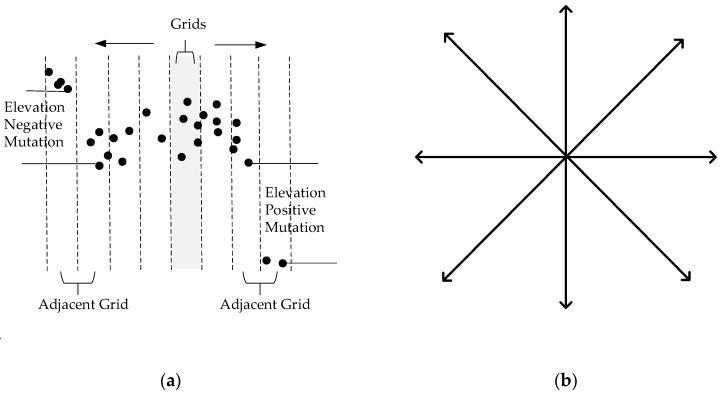
Altitude jump. (**a**) front view; (**b**) top view.

As shown in Figure 3a, the height difference of the lowest points of the adjacent grids is calculated along the straight line to both sides, with the grid having the points to be classified as the center. When the altitude difference is greater than a certain threshold value and the distance to the central grid is smaller than a certain threshold, altitude jump will be deemed to exist. If the altitude difference value is positive, it is a positive jump, and is calculated as 1. If the altitude difference value is negative, it is a negative jump and is calculated as −1. Finally, the sum of the absolute value of all jump values on eight directions evenly distributed from 0 to 360 degrees (Figure 3b) is computed as the final altitude jump. In this paper, we calculate a jump as 1 no matter whether it is a positive jump or a negative jump. The reason we distinguish between 1 and −1 is for the further optimization and expansion of the algorithm, such as counting the number of positive jumps and negative jumps separately.

#### 2.2.2. 3D Grid Features

Linear coefficient ($L_{1}$): The power line hangs naturally through the power tower, so the power line is distributed along a straight line when projected onto the horizontal plane. Based on this, a straight line is fitted, and the distances between various neighboring points to this straight line are calculated. The number of points whose distance is smaller than the threshold is calculated, and the ratio to the total number of neighboring points represents the linear coefficient. The value of the linear coefficient indicates the possibility that the point cloud forms a linear distribution in the local area.

Planar coefficient ($P_{1}$): When calculating the plane coefficient, the points in adjacent three-dimensional space are first fitted to a three-dimensional space plane, and then the distance from each neighboring point to the plane is calculated. The number of points whose distance is smaller than the threshold is calculated, and the ratio to the total number of neighboring points is the planar coefficient. The value of the planar coefficient indicates the possibility that the point cloud forms a planar distribution in a local area.

Linearity ($L_{2}$) and planarity ($P_{2}$). The covariance matrix of the three-dimensional coordinates of the point cloud in the neighborhood is calculated, and the eigenvalue relation of this matrix can be used to represent the three-dimensional space distribution of the point cloud in the neighborhood. The linearity and planarity are calculated through the eigenvalue of the matrix [22].

## 3. Classification of Power Facilities Based on Topological Constraints

The power facilities that are the subject of power line inspection are mainly the power lines and power towers in the transmission corridors. The other main types in transmission corridors are buildings, vegetation, and ground. Different objects in the power transmission corridor have different spatial morphological features. For instance, the power line always presents a linear distribution, and the power line is overhead. Surface points of construction show a planar distribution in a local range, and altitude jump exists in several directions with respect to ground points.

### 3.1. Feature Selection via Adaboost and Model Training

**Figure 4:**
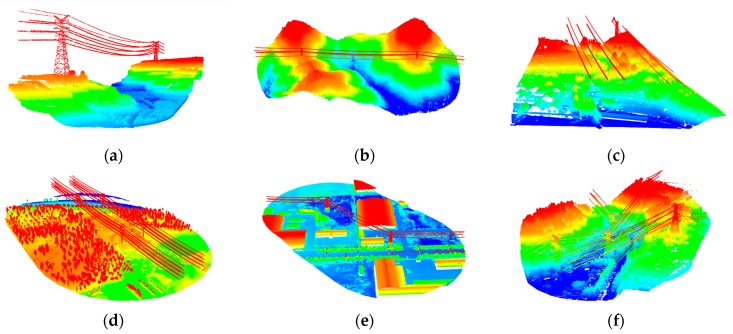
Training data of different terrains: (**a**) valley; (**b**) dense vegetation; (**c**) terraced field; (**d**) plain with sparse forest; (**e**) plain with the building area; and (**f**) steep forests with abundant vegetation.

Adaboost is a simple and useful machine learning algorithm. Its core idea is to combine weak classifiers with different weights into a strong classifier. The basic flow of point cloud classification using the Adaboost algorithm is: (1) data preprocessing; (2) feature calculation; (3) feature model training; and (4) classification based on the trained model. The Adaboost algorithm can be used to self-adaptively calculate the weights of different weak classifiers, namely, the importance of different features for classification of a certain ground object type. In order to verify the sensitivity of different objects in the power transmission corridor to the above features, a significant amount of point cloud data of the power transmission corridor is used to conduct classification training for different objects with the Adaboost algorithm, including data from plain areas, mountain areas, high mountain areas with steep terrain, building areas, dense vegetation areas, and terraced field areas. Some of the data we used are displayed in Figure 4.

The parameters used for feature extraction are set according to Reference [16], and are as follows: the grid length and width (a and b in Figure 2) are 0.75 m respectively; the slice thickness (c in Figure 2) is 0.75 m; and the radius of the local three-dimensional space neighborhood is 2 m. The top five features with the highest weight for different objects in the training results are selected, which are shown in Table 1. According to the training results, the point cloud classification of power lines has distinct features, but vegetation points and power tower points have the same key features. Therefore, by using the existing features, the Adaboost algorithm can well classify the power line points, but is unable to clearly distinguish power tower points from vegetation points, which is consistent with the conclusion of Reference [16]. In order to increase the classification accuracy of vegetation and power tower points, new features should be added. However, the increase in the number of features may not improve the classification performance of vegetation and power towers, and will lead to a reduction in the computational efficiency. In this paper, the topological relationship between power lines and power towers is utilized for constraint classification in order to increase the classification accuracy of power towers without adding new features.

### 3.2. Classification of Power Line Point Cloud

#### 3.2.1. Coarse Classification of Power Line Points

Power lines have obvious features, so the training model of the Adaboost algorithm performs well for the classification of power line points. However, there are still some wrongly classified points in the coarse result. The main reason for the misclassification of power line points is the calculation of its key features, which is greatly affected by the scale used for calculating these features.

#### 3.2.2. Fine Classification of Power Line Points

After the coarse classification of power line points, missing points and wrong points should be checked and corrected. The power lines with different voltage classes have different split states: single, 2-bundle, 4-bundle, 6-bundle, 8-bundle, etc. Power lines with different split states often present different characteristics at different local spatial scales. It is difficult to apply a fixed neighborhood spatial scale to power lines with multiple split states, so it is necessary to automatically select the optimal spatial scale for different split states when calculating features. According to the regulation of power line spacing specifications under the voltage class of 50–750 kV, five scales (1.2 m, 2.2 m, 3.2 m, 5.2 m, 7.2 m, 10.2 m, and 12.2 m) are used. By using linear features as the criterion, $L_{1}$ and $L_{2}$ under different scales are calculated, and the scale corresponding to the maximum product of $L_{1}$ and $L_{2}$ is selected as the optimum scale of the power line.

The core idea of the detection and optimization of power line points is to determine whether a certain point is on the power line. By searching for and identifying points on the power line along its direction, missing points will be found, and the wrong points will be corrected. Table 2 gives the pseudo-code of the refinement process of the coarse power line classification.

### 3.3. Power Tower Classification Based on Topological Relation

It is difficult to distinguish power tower points from vegetation points using the selected key features due to their overlap. In this paper, the topological relationship between power lines and power tower is utilized to classify power tower and vegetation points.

#### 3.3.1. Determination of Potential Areas of Power Towers Based on Topological Relationship

**Figure 5:**
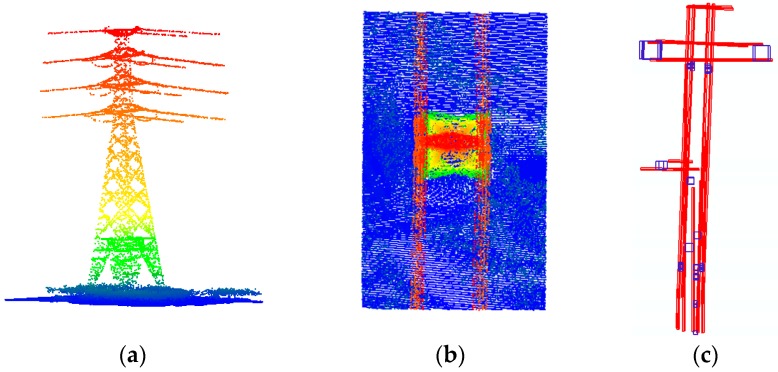
Potential power tower areas detection: (**a**) front view of a typical power tower; (**b**) top view of a typical power tower; (**c**) detected potential power tower areas, where the power lines are marked red, and the potential power tower areas are marked blue.

The power line is laid overhead through the power tower and suspended from the left and right side of the power tower or through the middle of the power tower. Based on this, the power lines and power towers have the following topological relationship: (1) When the power line is a single line, the power line passes through the middle of the tower head or hangs on a single crossarm; (2) when the power lines appear in pairs on both sides, the power tower is located in the middle position of two power lines, and the tower width is consistent with the width of the two power lines; and, (3) the height of some points in the power tower is consistent with the power line points hanging on it. A typical power tower is shown in Figure 5a,b.

Using the topological relationship between the power towers and power lines, potential areas of the power towers can be determined. The specific steps are as follows:

1. Determination of single-sided power transmission lines: The right and left sides of the transmission line are determined, and each power line after classification is projected onto the plane. The power lines whose direction are approximate, and the distance between them is less than a certain threshold r is classified as one side of the power line, and the center line of the 2D bounding box from the top view is taken as the line along the power line, as shown in Figure 5c. In this paper, r is set as 3 m.
2. Matching the right and left side power lines to determine the corresponding left and right sides of each level: Using the highest point corresponding to each line, the two lines whose direction and elevation are close to each other and whose distance is the smallest are taken as the left and right sides of each power line.
3. Determination of potential areas of power towers by key features: By constraining the right and left sides of each level of the power line, the points lying between the left and right power lines are determined. If the topological conditions are met, the potential areas of the power tower points can be determined by setting the distance between two lines as the length of a side. The results of the potential areas selected are presented as small blue squares in Figure 5c.

#### 3.3.2. Power Tower Classification under Constraints in Potential Areas

The points in the potential areas are classified to find the power tower points. The key features of the point cloud in the region are calculated and classified according to the classification model in the following two steps:

1. Determination of the optimum scale for key features of the power tower: The ranges of different areas vary, and the sizes of power towers at various levels are different. Therefore, the side length of the area to be selected is adopted as the scale of feature calculation in this area, to make it robust at different high voltage levels.
2. Power tower classification based on the Adaboost algorithm and topological relationship: According to different terrain types, the corresponding features are selected based on Table 1, and the power towers in each region are classified considering the classification model. Then, the topological condition (3) in Section 3.3.1 of the same elevation between the power towers and the power line points on the towers are added to further improve the classification accuracy of the power towers.

## 4. Experiments and Discussion

**Figure 6:**
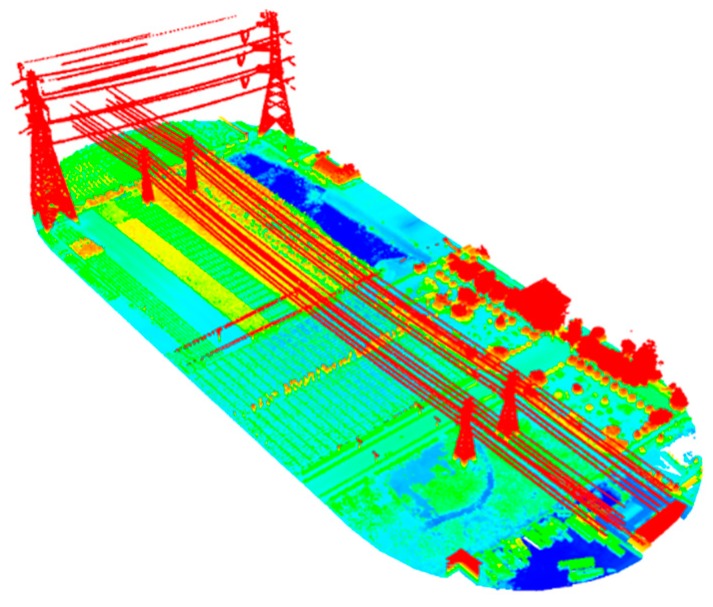
Point cloud data.

The experimental data were from Zhuozhou, Hebei Province, China, and were obtained by UAV LiDAR. It includes not only 550 kV and 110 kV high-voltage overhead transmission facilities, but also low-voltage 380 V distribution lines, thereby making it more challenging to classify. The number of points of the experimental point cloud is 4,688,429, and the point cloud density is 51.36/m^2^, as shown in Figure 6 (rendered according to the altitude).

In order to verity the effectiveness of the proposed algorithm, the classification result of manual detection is used as reference data, which was conducted carefully using CloudCompare [23] software. In addition, to visualizing the performance, we use a precision–recall curve for quality evaluation, which is defined as:

(1) $$\mathrm{Precision}=\;\frac{\mathrm{TP}}{\mathrm{TP}+\mathrm{FP}},$$

(2) $$\mathrm{Recall}=\frac{\mathrm{TP}}{\mathrm{TP}+\mathrm{FN}},$$

(3) $$F_{1}\;\mathrm{Score}\;=\frac{2*\mathrm{Precision}*\mathrm{Recall}}{\mathrm{Recision}+\mathrm{Recall}},$$

where TP, FP, and FN are true positive, false positive, and false negative corresponding to ground truth, respectively. Precision reflects the correctness of points that are classified to a certain type, recall reflects the ability of finding a certain type, and the F_1_ score is the comprehensive evaluation of precision and recall.

The experiments were run using C++ in a Windows 10 environment with a PC with a 3.7 GHz CPU and 64 GB of RAM. To improve the running speed, we used multi-thread computing, and the running time for the considered point cloud dataset was 23 s.

### 4.1. Power Transmission Line Extraction under Different Scales

**Figure 7:**
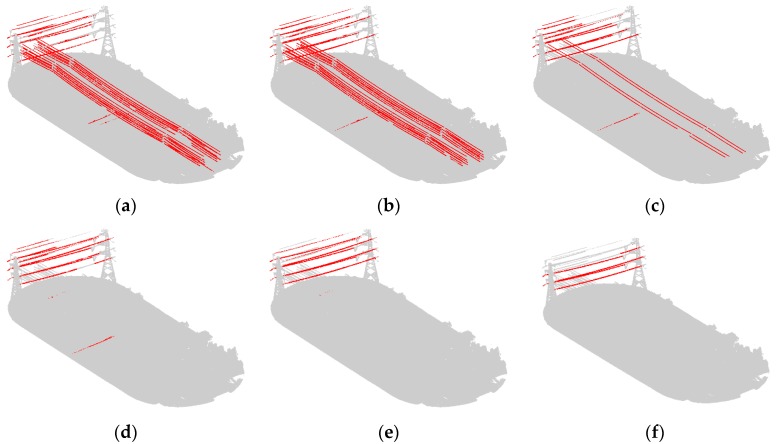
Power line classification visualization results under different scales: (**a**) 2.2 m; (**b**) 3.2 m; (**c**) 5.2 m; (**d**) 7.2 m; (**e**) 10.2 m; and (**f**) 12.2 m.

**Figure 8:**
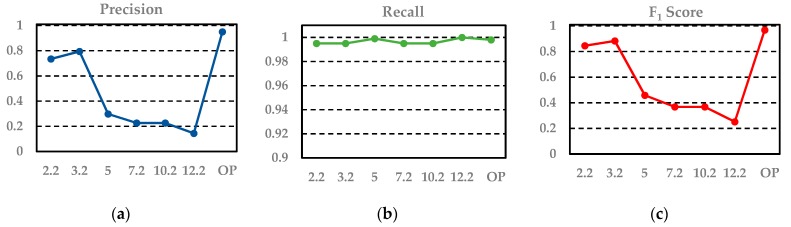
Precision–recall curve under different scales (**a**–**c**).

**Figure 9:**
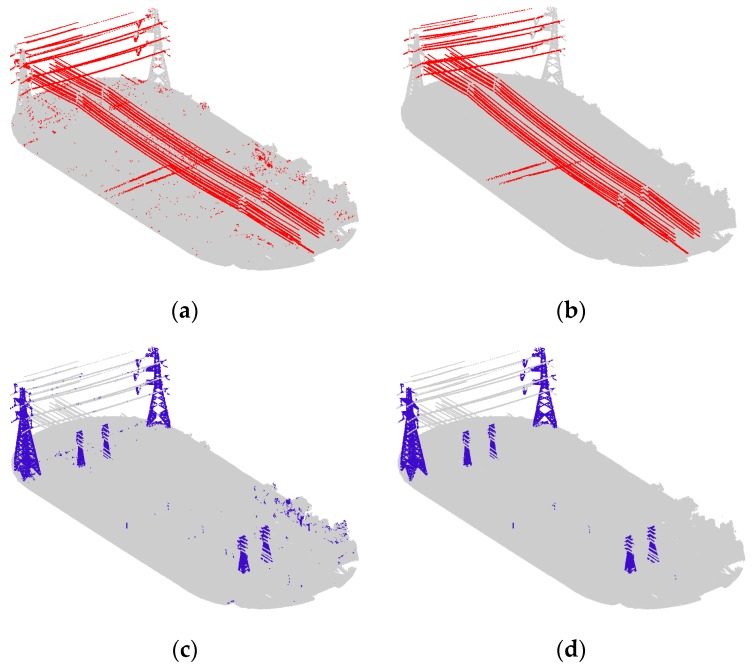
Classification results: (**a**) coarse result of power line; (**b**) fine result of power line; (**c**) coarse result of power tower; and (**d**) fine result of power tower.

In order to explore the effect of scale on power transmission line extraction and to prove the effectiveness of the optimum scale selection strategy, we experimented using the scales of 2.2 m, 3.2 m, 5.2 m, 7.2 m, 10.2 m, and 12.2 m, and the optimum scale, separately. Figure 7 and Figure 9b display the visualization results with classified power transmission line points marked in red and the other points marked in gray; Figure 9b is the result under the optimal scale. Figure 8 displays the change in the values of precision, recall, and F_1_ score under different scales.

From the results, we can see that the coarse result has many noisy points. When applying a fixed scale, the classified power transmission line points always maintain a high precision because the key features we selected are distinct. Furthermore, after the combination with proper weights using the Adaboost algorithm, the classifier has better discrimination ability. However, the value of recall is low and varied because power transmission lines with different voltages correspond to different optimum scales. Thus, when the selected scale is similar to the optimum scale of a certain voltage, this portion of power transmission line points will be found. Moreover, if these points account for a relatively high percentage of all power transmission line points, the recall value will be large; otherwise, it will be small. Differently from a fixed scale, our optimum scale selection strategy can find the optimum scales for different voltages; thus, the power transmission line points of different voltages are all found. As a result, the values for precision, recall, and F_1_ score of the proposed algorithm are all the highest.

### 4.2. Comparation of Coarse and Fine Results of Power Lines and Power Towers

The power line and power tower classification results obtained directly from the training model with the Adaboost algorithm were taken as coarse results, and the improved results using the method described in Section 3.2 and Section 3.3 were taken as fine results. The visualization results are displayed in Figure 9, while the results accuracy is shown in Table 3.

From Figure 9, we can see that the noisy points were eliminated and the correct points were retained. In addition, some wrong points were reclassified to the correct category. Especially, the precision of the power tower improved from 0.637 to 0.902, the F_1_ score of power lines increased by 3.6%, while the F_1_ score of power towers increased by 17.2%. The reason why the precision of the power tower improved so much is the use of topological constraints between the power lines and the power towers. As a result, the misclassification of vegetation points and power tower points is largely reduced, enabling the extraction of power towers of different voltage levels.

### 4.3. Overall Evaluation

**Figure 10:**
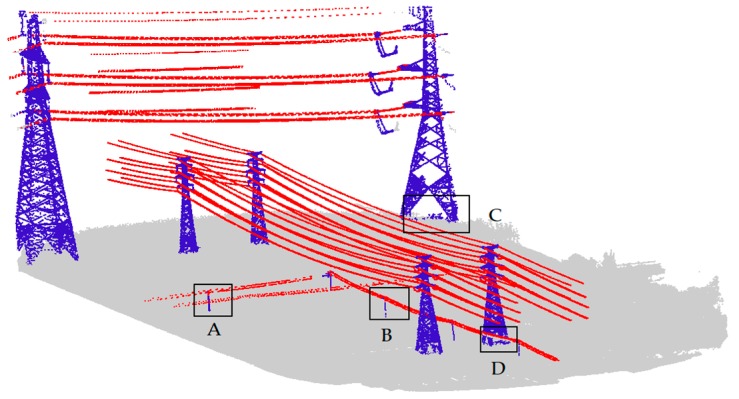
Final classification of the point cloud data.

**Figure 11:**
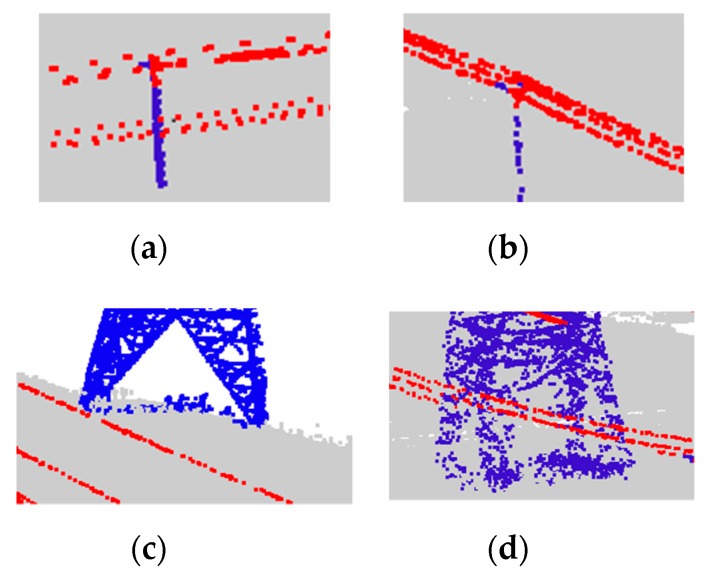
A zoomed-in view of the specific areas in Figure 10: (**a**) area A; (**b**) area B; (**c**) area C; and (**d**) area D.

The overall classification results of power facilities are shown in Figure 10, including three types of power lines, power towers, and non-power facilities. Figure 11 gives a close view of four places that are typically hard to classify.

From Figure 10, we can see that the proposed algorithm has good adaptability to different voltage levels of transmission facilities, high classification accuracy, and high classification robustness in a complex power scenario, especially for the low-voltage facilities. For example, in the areas of Figure 11a,b, the power lines and power towers were effectively classified.

However, there are still some places where the points were not classified correctly, such as the areas presented on Figure 11c,d, where a small portion of power tower points was not correctly classified, which were mainly concentrated at the bottom of the power tower. The reason is that the number of points in that area is small, and it is too close to vegetation to distinguish them effectively.

### 4.4. Robustness Test

**Figure 12:**
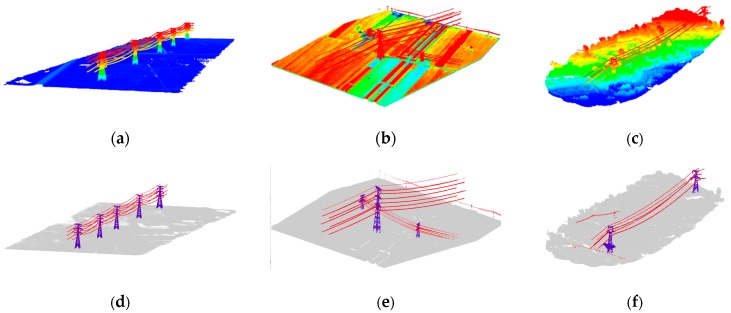
Robustness test: (**a**) data of test 1; (**b**) data of test 2; (**c**) data of test 3; (**d**) classification result of test 1; (**e**) classification result of test 2; and (**f**) classification result of test 3.

In order to test the robustness of the proposed method, we selected three typical situations. Test 1 and test 2 are both located in the plane area, where test 1 has fewer power towers, while the power lines cross each other in test 2. Test 3 presents a mountain area, having a lot of vegetation which surrounds the power towers, and multi-scale power lines may be found there as well. The results are displayed in Figure 12 and Table 4.

From the results, we can see that the proposed algorithm performs well in test 1 and test 2. For test 3, most of the power line points are correctly classified, but the classification accuracy of the power tower is relatively low. The reason is that the bottom points of the power tower are mixed with the vegetation points, and the training data are located primarily in plane areas. Thus, the result could be improved by adding more samples of the mountain area to the training data.

### 4.5. Discussion

Point cloud data have relatively strong spatial divergence, and the identification of an effective space neighborhood scale is the key to calculate data features. In this paper, linear key features are utilized to choose the optimum scale for point clouds in different areas, and feature extraction is conducted on the basis of the optimum scale. Point clouds classification is conducted under the support of a classification model obtained from the Adaboost algorithm. Due to the high similarity of the features of power towers and vegetation, it is difficult to distinguish power tower points from vegetation using only the selected features. Thus, we added the topological relationship between power towers and power transmission lines. As a result, the classification accuracy of power tower points was improved without adding more features.

Since there is no benchmark for the classification of power facilities point clouds, it is hard to accurately compare our approach to other methods. References [15,16,24] all performed well in power facility classification, and also used machine learning methods, where Reference [15] and Reference [24] extracted 21 features and Reference [16] extracted 26 features. Compared to these methods, we only use five features with the best discrimination for each kind of object, and the topological constraint optimization is fast, so our method is expected to be much faster than the other methods. In addition, the proposed algorithm maintains high accuracy in a scenario where different voltage levels exist simultaneously in power lines, whereas other methods did not consider this kind of situation.

## 5. Conclusions

Based on the machine learning algorithm Adaboost, this paper conducted a feature model classification of power facilities under the constraints of topological relationship. The Adaboost algorithm can produce high-quality classification results, but there are still some errors in the coarse classification because the features extracted cannot adequately reflect the whole scene. Moreover, some errors are produced during the feature extraction process because different objects have different optimal scales. To address the problem caused by a non-optimal scale, we propose an adaptive algorithm to find the optimal scale, improving the accuracy of feature extraction. By utilizing the topological relationship between power lines and power towers, wrongly classified points are re-classified correctly, and missing points are found. Experiments show that a comparatively good classification result is gained without the need of many features.

In general, the proposed method can be applied well in most areas where complete point clouds of power facilities can be obtained. However, in areas with large fluctuations and dense trees, low penetration of the laser leads to fewer laser points on the ground, which has a great impact on the accuracy of the features, reducing the final classification accuracy. Special research should be carried out on the classification of power lines in this kind of scenario.

In the future, the classification of overhead power transmission lines at corners and at the connections of lines and towers will be further studied to improve the classification accuracy of power lines. In addition, the classification of other objects in the electric corridor will be added to realize the automatic analysis of power line patrols in the power transmission corridor.

## Figures and Tables

**Table 1:** Key features selected for different objects.

Type	$N_{1}$	$N_{2}$	$V_{1}$	$V_{2}$	$H_{1}$	$S_{1}$	$L_{1}$	$P_{1}$	$L_{2}$	$P_{2}$
Power Line			√	√	√		√		√	
Power Tower	√	√		√	√	√				
Vegetation	√	√		√	√	√				
Ground Point	√		√	√				√		√
Construction	√			√		√		√		√

**Table 2:** Pseudo-code of the refinement process of coarse power line classification.

**Input:** coarse power line classification result $PC_{in}$	
**Output**: refined power line classification result $P_{fine}$	
1	**for** All the points in $PC_{in}$, **do**
2	Put the points labeled power line into $P_{coarse}$ and initialized as unused
3	**for** each $p\in P_{coarse}$, and p is marked unused, **do**
4	Select the optimal scale $S_{optimal}$
5	Search the neighborhood $M_{p}$ of p based on $S_{optimal}$
6	Use $M_{p}$ to fit a line line
7	Set threshold as $0.5*\;S_{optimal}$
8	Calculate the average distance $d_{ave}$ of each point in $M_{p}$ to line
9	**if** $d_{ave}$ is smaller than threshold, **do**
10	Reclassify $M_{p}$ as power line point, and Mark $M_{p}$ as used
11	Calculate the end points p_front, p_back of line
12	**for** $p_{front},\;p\_back$, **do**
13	Search the neighborhood $M_{pp}$ of p based on $S_{optimal}$
14	Calculate the maximum distance d_max for each point in $M_{pp}$ to line
15	**if** d_max is smaller than threshold, **do**
16	Reclassify $M_{pp}$ as power line point;
17	Mark $M_{pp}$ as used
18	Combine $M_{pp}$ into $M_{p}$
19	Re-fitting line line by using $M_{p}$
20	Go into step 11
21	**else if** d_max is bigger than threshold, **do**
22	Classify the points whose distance is smaller than threshold as power line point, the others as un-power line point
23	Mark $M_{pp}$ as used
24	**if** all the points are marked used, **do**
25	Put all the points marked power line point into $P_{fine}$
26	**Return** $P_{fine}$

**Table 3:** Classification accuracy statistics of power facilities.

Classification Result	Precision	Recall	F_1_ Score
Coarse Result of Power Line	0.900	0.967	0.932
Fine Result of Power line	0.988	0.950	0.969
Coarse Result of Power Tower	0.637	0.958	0.765
Fine Result of Power Tower	0.902	0.968	0.934

**Table 4:** Classification accuracy statistics of test data.

Result		Test 1	Test 2	Test 3
Power Line	Precision	0.998	0.951	0.952
	Recall	0.968	0.994	0.982
	F_1_ Score	0.982	0.972	0.967
Power Tower	Precision	0.952	0.990	0.550
	Recall	0.996	0.881	0.963
	F_1_ Score	0.974	0.933	0.701
